# Detecting early cardiomyopathy in transthyretin variant carriers: reappraising the diagnostic value of Perugini grade 1 radiotracer uptake on bone scintigraphy

**DOI:** 10.1007/s00259-025-07328-6

**Published:** 2025-05-23

**Authors:** H. S. A. Tingen, M. Berends, A. Tubben, P. van der Meer, R. H. J. A. Slart, J. Bijzet, P. A. van der Zwaag, C. Kimmich, C. Knackstedt, F. L. H. Muntinghe, E. J. Houwerzijl, B. P. C. Hazenberg, H. L. A. Nienhuis

**Affiliations:** 1 Department of Nuclear Medicine and Molecular Imaging, Groningen Amyloidosis Centre of Expertise, Groningen University, University Medical Centre Groningen, Groningen, The Netherlands; 2 Department of Internal Medicine, Groningen Amyloidosis Centre of Expertise, Groningen University, University Medical Centre Groningen, Groningen, The Netherlands; 3 Department of Cardiology, Groningen Amyloidosis Centre of Expertise, Groningen University, University Medical Centre Groningen, Groningen, The Netherlands; 4 Biomedical Photonic Imaging Group, Faculty of Science and Technology, University of Twente, Enschede, The Netherlands; 5 Department of Rheumatology & Clinical Immunology, Groningen Amyloidosis Centre of Expertise, Groningen University, University Medical Centre Groningen, Groningen, The Netherlands; 6 Department of Clinical Genetics, Groningen Amyloidosis Centre of Expertise, Groningen University, University Medical Centre Groningen, Groningen, The Netherlands; 7 Department of Oncology and Haematology, Klinikum Oldenburg, University Clinic, Oldenburg, Germany; 8 Department of Cardiology, Cardiovascular Research Institute Maastricht (CARIM), Maastricht University, Maastricht, The Netherlands

**Keywords:** ATTRv, Screening, ATTR-CM, Diagnosis, Early detection

## Abstract

**Purpose:**

To determine whether *TTRv* carriers with Perugini grade 1 cardiac radiotracer uptake on [^99m^Tc]Tc- hydroxydiphosphonate bone scintigraphy have or develop ATTR-CM.

**Methods:**

This retrospective observational study was conducted at the Groningen Amyloidosis Centre of Expertise between April 2012 and June 2023. *TTRv* carriers with Perugini grade 1 uptake on bone scintigraphy were followed until to June 2024. Data on symptoms, biomarkers, imaging, and biopsies were collected. A descriptive analysis was performed to evaluate whether carriers met the diagnostic criteria for ATTR-CM or ‘probable ATTR-CM’ at baseline and follow-up.

**Results:**

Out of 178 *TTRv* carriers in screening, 12 carriers had Perugini grade 1 cardiac radiotracer uptake on bone scintigraphy. At baseline, 2 carriers met the diagnostic criteria for ATTR-CM and 3 carriers met the criteria for probable ATTR-CM. Of the 7 carriers without (probable) ATTR-CM at baseline, 3 carriers were diagnosed with ATTR-CM during follow-up and 1 carrier developed probable ATTR-CM during follow-up. Three carriers showed signs of cardiomyopathy during follow-up, but did not meet the criteria for (probable) ATTR-CM. One of these cases may have been false-positive due to hydroxychloroquine use.

**Conclusion:**

Our findings suggest that Perugini grade 1 cardiac radiotracer uptake is an early marker of ATTR-CM in *TTRv* carriers, potentially enabling earlier diagnosis and intervention.

## Introduction

Individuals with a pathogenic transthyretin gene variant (*TTRv*) are at risk for developing hereditary transthyretin (ATTRv) amyloidosis [1]. In this population, screening for ATTR cardiomyopathy (ATTR-CM), a common disease manifestation, is essential for the early detection of subclinical ATTR-CM and timely treatment initiation, which improves patient outcomes [2, 3].

Bone scintigraphy has high accuracy for detecting ATTR-CM [4, 5]. Its high accuracy and non-invasive nature make bone scintigraphy an attractive screening tool for ATTR-CM. Perugini grade 2 or 3 cardiac radiotracer uptake is diagnostic for ATTR-CM, provided there is no evidence for immunoglobulin light chain amyloidosis. In contrast, grade 1 uptake requires histological confirmation and additional findings on cardiac imaging to diagnose ATTR-CM [6]. However, in high-risk individuals such as *TTRv* carriers, it may represent an early indicator of ATTR-CM.

We investigated whether *TTRv* carriers with Perugini grade 1 cardiac radiotracer uptake on [^99m^Tc]Tc- hydroxydiphosphonate bone scintigraphy have or develop ATTR-CM. The study aimed to determine if Perugini grade 1 could be considered diagnostic of early-stage ATTR-CM in *TTRv* carriers.

## Methods

This retrospective observational study included all *TTRv* carriers undergoing treatment or screening at the Groningen Amyloidosis Centre of Expertise (GrACE) between April 2012 and June 2023 and was approved by the ethical board of the University Medical Centre Groningen (registration number: 17471). As a part of the GrACE screening program for ATTR-CM, all *TTRv* carriers regularly underwent bone scintigraphy. The frequency of screening was on average every 3 years; however, the interval varied within and between patients and was determined based on disease characteristics.

Bone scintigraphy was performed 3 h after administration of 450 to 750 MBq [^99m^Tc]Tc-hydroxydiphosphonate on dedicated single photon emission computed tomography (SPECT)/CT systems (Symbia T2, Symbia T16 or Symbia Intevo, Siemens Healthineers, Erlangen, Germany) equipped with a low-energy high-resolution collimator. Anterior planar whole-body scans were scored according to the Perugini grading system and SPECT/CT scans were reviewed to confirm myocardial uptake [7].

For patients with Perugini grade 1 uptake on bone scintigraphy, data were collected from patient records until June 2024, including symptoms, cardiac biomarkers, abdominal fat tissue aspirates and tissue biopsies for amyloid detection, electrocardiography, echocardiography, cardiac magnetic resonance imaging (CMR), and bone scintigraphy. The first bone scintigraphy showing Perugini grade 1 uptake was considered the baseline scan. A descriptive analysis was performed to evaluate whether individuals met the diagnostic criteria for ATTR-CM, as outlined in the European Society of Cardiology (ESC) position paper, at baseline and follow-up [6]. *TTRv* carriers who did not meet the ESC diagnostic criteria were classified as having ‘probable ATTR-CM’ if they met the criteria previously proposed by Rapezzi et al. and later adjusted by Klaassen et al. [8, 9]. These criteria include increased wall thickness on echocardiography (end-diastolic interventricular septal and/or left ventricular posterior wall thickness ≥ 12 mm) and/or advanced atrioventricular block (greater than first-degree) or intraventricular conduction disturbances (bundle branch blocks/hemiblocks) on electrocardiography without an alternative explanation, combined with histologically confirmed amyloid in an extracardiac biopsy.

## Results

**Fig. 1:**
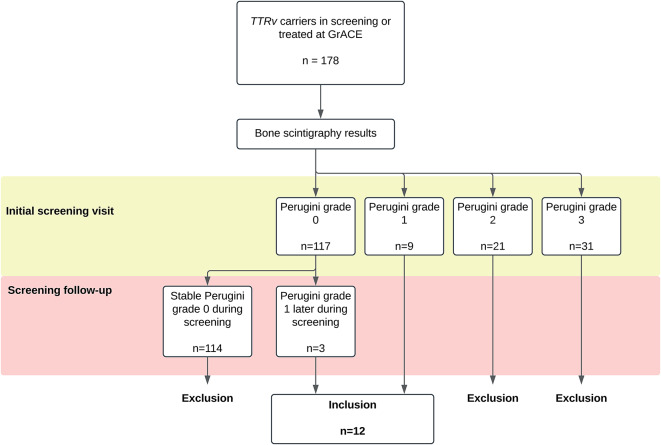
Flow chart of inclusion. TTRv = transthyretin gene variant; GrACE = Groningen Amyloidosis Centre of Expertise

**Fig. 2:**
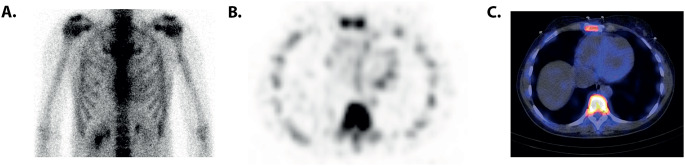
Bone scintigraphy showing Perugini grade 1 cardiac radiotracer uptake in an ATTRv amyloidosis patient with the p.(Ser43Asn) variant. **A**: anterior planar scintigraphy **B**: axial SPECT, and **C** axial SPECT/CT fusion. ATTRv = hereditary transthyretin; SPECT = single-photon emission tomography; CT = computed tomography

**Fig. 3:**
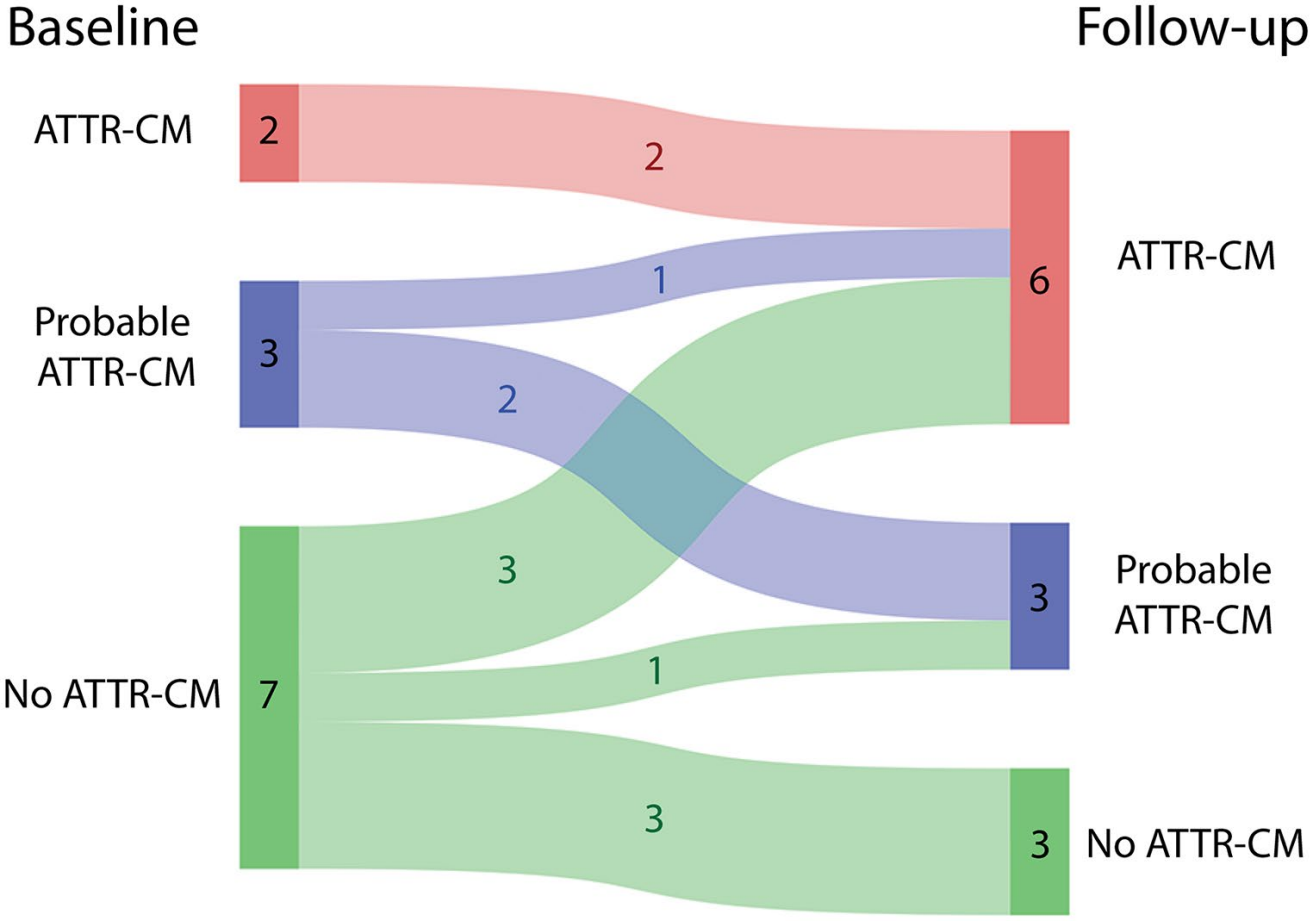
Sankey plot showing the study category at baseline and follow-up for each patient. ATTR-CM = transthyretin amyloid cardiomyopathy

A total of 178 *TTRv* carriers were screened between 2012 and 2023. Among them, 117 carriers had Perugini grade 0 (66%), 9 had grade 1 (5%), 21 had grade 2 (12%), and 31 had grade 3 (17%) cardiac radiotracer uptake on the first bone scintigraphy during screening. Notably, 3 of the 117 *TTRv* carriers with Perugini grade 0 uptake on their initial bone scintigraphy showed Perugini grade 1 uptake on a follow-up scan (Fig. 1). Clinical data were collected for the 12 *TTRv* carriers with Perugini grade 1 uptake on bone scintigraphy at any time during screening. A representative case is shown in Fig. 2. Their *TTR* gene variants were p.(Val50Met) (*n* = 7), p.(Glu109Lys) (*n* = 2), and p.(Ser43Asn) (*n* = 1), p.(Ile127Val) (*n* = 1), and p.(Val114Ala) (*n* = 1). Table 1; Fig. 3 provide information on whether these *TTR*v carriers met the criteria for (probable) ATTR-CM at the baseline scan or during follow-up, along with relevant clinical parameters and details.

**Table 1:** Fulfilment of criteria for (probable) ATTR-CM and clinical parameters in *TTRv* carriers with perugini grade 1 cardiac Radiotracer uptake

Pt	Study category	TTRv	First proof of amyloid in tissue	Time point	ATTR-CM	Year	Clinical parameters							
							Treatment	ESC echo and CMR criteria	Perugini grade	IVSt on echo (mm)	LVPWt on echo (mm)	Conduction disturbances as defined in main text	NTproBNP (ng/L)	Hs-cTnT (ng/L)
1	Diagnosis at baseline	p.(Ile127Val)	Baseline *Fat aspirate*	B	Diagnosis	2020	None	Yes	1	14	12	No	1063	26
2	Diagnosis at baseline	p.(Val114Ala)	Baseline *Fat aspirate*	B	Diagnosis	2022	None	Yes	1	12	12	No	71	12
3	Diagnosis during FU	p.(Ser43Asn)	Baseline *Fat aspirate*	B	Insufficient evidence	2017†	St	No	1	8	8	No	80	5
				FU	Diagnosis	2020	St, Si	No	2	8	9	No	63	8
4	Diagnosis during FU	p.(Val50Met)	No amyloid	B	Insufficient evidence	2012	None	No	1	13	10	No	30	10
				FU	Diagnosis	2016	None	No	2	13	8	No	63	18
5	Diagnosis during FU	p.(Val50Met)	Baseline *Fat aspirate*	B	Insufficient evidence	2012	St	No	1	11	10	No	247	16
				FU	Diagnosis	2014	St	No	2	13	13	No	333	17
6	Probable ATTR-CM at baseline, diagnosis during FU	p.(Glu109Lys)	Baseline *GI biopsy*,* fat aspirate*	B	Probable	2012	St	No	1	14	10	No	86	6
				FU	Diagnosis	2016	St, Si	No	2	15	10	No	102	8
7	Probable ATTR-CM at baseline	p.(Val50Met)	Baseline *Fat aspirate*	B	Probable	2021†	OLTx	No	1	14	10	Yes	572	16
				FU	Probable	2022	OLTx	No	1	14	10	Yes	544	19
8	Probable ATTR-CM at baseline	p.(Val50Met).	Baseline *Fat aspirate*	B	Probable	2012	None	No	1	12	13	Yes	547	41
				FU	Probable	2015	None	No	1	12	14	Yes	1547	91
9	Probable ATTR-CM during FU	p.(Val50Met)	During follow-up *Fat aspirate*	B	Insufficient evidence	2013	None	No	1	10	10	No	105	12
				FU	Probable	2024	Si	No	1	10	10	No	522	19
10	No diagnosis	p.(Val50Met)	No amyloid	B	Insufficient evidence	2020	None	No	1	15	9	No	79	8
11‡	No diagnosis	p.(Glu109Lys)	During follow-up *Fat aspirate*	B	Insufficient evidence	2015†	St	No	1	8	8	No	623	< 3
				FU	Insufficient evidence	2023	St	No	1	8	10	No	433	9
12	No diagnosis	p.(Val50Met)	Baseline *Nerve biopsy*,* fat aspirate*	B	Insufficient evidence	2015	St	No	1	11	10	No	54	9
				FU	Insufficient evidence	2022	St, Si	No	1	9	11	No	87	11

Follow-up data are presented up to the time of diagnosis, although follow-up may have continued thereafter. For *TTRv* carriers with only a single bone scintigraphy, follow-up data from other tests are not included but may be available for assessment of cardiac status

FU = follow-up; Pt = patient; *TTRv* = transthyretin gene variant; B = baseline; ESC = European Society of Cardiology; CMR = cardiac magnetic resonance imaging; IVSt = inter ventricular septal thickness; NTproBNP = N-terminal pro b-type natriuretic peptide; hs-cTnT = high sensitivity cardiac troponin T; St = transthyretin stabilizer; Si = transthyretin gene silencer; OLTx = orthotopic liver transplantation; ATTR-CM = transthyretin amyloid cardiomyopathy; GI = gastro-intestinal; † = these carriers had a previous bone scintigraphy showing Perugini grade 0; ‡ = this carrier used hydroxychloroquine

At baseline, 2 out of 12 *TTRv* carriers met the ESC diagnostic criteria for ATTR-CM based on extracardiac biopsy and echocardiography. Three others were classified as having probable ATTR-CM at baseline. One of these carriers met the ESC diagnostic criteria after a follow-up of 3.5 years and the other 2 carriers showed worsening of cardiac parameters over follow-up periods of 4 and 12 years. Of the 7 carriers without (probable) ATTR-CM at baseline, 3 carriers were diagnosed with ATTR-CM, after a follow-up of 2, 2 and 4 years, based on Perugini grade 2 cardiac radiotracer uptake on bone scintigraphy. Of the remaining 4 carriers, 1 carrier developed probable ATTR-CM after a follow-up of 11 years, based on increased wall thickness on echocardiography and a positive fat tissue aspirate. Three carriers showed signs of cardiomyopathy during follow-up, but did not meet the criteria for ATTR-CM or probable ATTR-CM. These signs included abnormal gadolinium kinetics on CMR, reduced global longitudinal strain and increased interventricular septal thickness on echocardiography. In 1 of these carriers, bone scintigraphy results might have been false-positive due to the use of hydroxychloroquine [6]. Moreover, it is important to note that treatment for ATTRv neuropathy was initiated during follow-up for all 3 of these carriers, potentially slowing or even halting the progression of ATTR-CM.

## Discussion

To our knowledge, this is the largest study to date that longitudinally evaluates whether *TTRv* carriers with Perugini grade 1 uptake on bone scintigraphy have or develop ATTR-CM. In 9 out of 12 *TTRv* carriers (75%), Perugini grade 1 cardiac radiotracer uptake was indicative of (probable) ATTR-CM or its development. In 2 out of 12 individuals (17%), signs of cardiac deterioration were observed during follow-up, but were insufficient to diagnose (probable) ATTR-CM. In the remaining patient, grade 1 uptake might have been false-positive due to hydroxychloroquine use [6].

Our data highlight that cardiac radiotracer uptake on bone scintigraphy is a dynamic phenomenon. In our cohort, 3 *TTRv* carriers initially showed no cardiac radiotracer uptake but later developed Perugini grade 1 uptake. Similarly, 4 carriers progressed from grade 1 to grade 2 uptake during follow-up. In line with our findings, Minutoli et al. previously reported a case series including 3 asymptomatic *TTRv* carriers who developed symptoms of ATTR-CM along with cardiac abnormalities, in whom Perugini grade 1 uptake preceded these changes [5]. Consistently, Rauf et al. showed that Perugini grade 1 uptake in ATTR-CM was associated with earlier disease stages compared to higher grades according to NT-proBNP levels, echocardiography and CMR [10]. In the context of these studies, our findings provide additional support that Perugini grade 1 uptake represents early-stage ATTR-CM in *TTRv* carriers.

Moreover, previous studies have shown that Perugini grade 1 is not an infrequent finding in ATTR-CM as it is observed in 8% of patients with endomyocardial biopsy-confirmed ATTR-CM [4]. In addition, the specificity of a positive bone scintigraphy for cardiac amyloidosis is high, ranging from 97 to 99% depending on the non-(cardiac) amyloidosis controls chosen [4]. Rauf et al. reported that of 3354 patients referred for suspected or proven cardiac amyloidosis, 183 had Perugini grade 1 uptake on bone scintigraphy, with 182 diagnosed with cardiac amyloidosis and only 1 false positive [10]. Further supporting the notion that *TTRv* carriers with Perugini grade 1 uptake on bone scintigraphy should be considered as having early-stage ATTR-CM.

Major limitations of our study are the absence of endomyocardial biopsies and incomplete echocardiographic and CMR data for some patients. Other limitations are the low number of patients with Perugini grade 1 radiotracer uptake and variability in follow-up intervals. Furthermore, the initiation of treatment in some individuals may have slowed or halted disease progression. All these limitations, related to the retrospective nature of this study, could potentially have led to an underestimation of the number of carriers with (probable) ATTR-CM.

This study is of particular importance in the context of the upcoming ACT-EARLY trial (NCT06563895), which evaluates prophylactic therapy for ATTR amyloidosis in asymptomatic *TTRv* carriers. Cases with Perugini grade 1 uptake are likely to be encountered during this trial, and endomyocardial biopsies will be performed in these cases to confirm diagnosis. This study may provide definitive proof of the diagnostic value of Perugini grade 1 radiotracer uptake in *TTRv* carriers.

Our findings, along with previous studies, suggest that Perugini grade 1 cardiac radiotracer uptake is an early marker of ATTR-CM in *TTRv* carriers, potentially enabling earlier diagnosis and intervention. Until further validation is available, Perugini grade 1 uptake should be considered strongly indicative of early-stage ATTR-CM in this population, provided known causes of false positives are excluded.

## Data Availability

The datasets generated during and/or analysed during the current study are available from the corresponding author on reasonable request.

## Abbreviations

ATTR-CM: Transthyretin Amyloid Cardiomyopathy
ATTRv: Hereditary Transthyretin Amyloidosis
CMR: Cardiac Magnetic Resonance Imaging
ESC: European Society of Cardiology
GrACE: Groningen Amyloidosis Centre of Expertise
NT-proBNP: N-terminal pro b-type Natriuretic Peptide
SPECT/CT: Single Photon Emission Computed Tomography/Computed Tomography
TTRv: Transthyretin Gene Variant
